# Management of anastrozole-induced bone loss in breast cancer patients with oral risedronate: results from the ARBI prospective clinical trial

**DOI:** 10.1186/bcr2565

**Published:** 2010-04-16

**Authors:** Christos Markopoulos, Evagelos Tzoracoleftherakis, Athanassios Polychronis, Basileios Venizelos, Urania Dafni, Grigorios Xepapadakis, John Papadiamantis, Vasilios Zobolas, John Misitzis, Kyriakos Kalogerakos, Angeliki Sarantopoulou, Nikolaos Siasos, Dimitrios Koukouras, Zoh Antonopoulou, Spyros Lazarou, Helen Gogas

**Affiliations:** 1 Hellenic Society of Breast Surgeons, 6 Eslin Street, Athens 11523, Greece; 2 Laboratory of Biostatistics, Department of Nursing, University of Athens, 123 Papadiamantopoulou Street, Athens 11527, Greece

## Abstract

**Introduction:**

The aim of this multicenter, phase III, prospective open label clinical trial was to investigate the effect of risedronate (R) on bone mineral density (BMD) in postmenopausal, early breast cancer (BC) patients scheduled to receive anastrozole (A).

**Methods:**

Pre-treatment BMD of 213 patients with hormone receptor-positive BC was evaluated at lumbar spine (LS) and hip (HP). Patients were categorized according to their baseline BMD T-score as being at low, moderate and high risk of osteoporosis. Low risk patients received anastrozole only (A), moderate risk were randomized to anastrozole +/- risedronate (A+/-R) administration and high risk patients received anastrozole + risedronate (A+R). Anastrozole was given at a dosage of 1 mg/day while oral risedronate was given at 35 mg/week. BMD was then assessed at 12 and 24 months. All patients received daily supplements of calcium (1000 mg/day) and vitamin D (400 IU/day).

**Results:**

At 24 months, in the moderate risk group, treatment with A+R resulted in a significant increase in BMD at LS and HP compared to treatment with A only (5.7% *v *-1.5%, Wilcoxon test *P* = 0.006, and 1.6% *v *-3.9% Wilcoxon test *P* = 0.037, respectively), while no significant difference was found at 12 months; 24.3% of the patients moved to normal BMD region. In the high risk group, a significant increase for LS was detected both at 12 and 24 months (6.3% and 6.6%, *P* < 0.001) but not for HP; BMD in 14% of patients improved to the osteopenic region. In the low risk group, a significant decrease of BMD was detected at 12 months for LS and HP (-5.3% *P* < 0.001 and -2.4% *P* < 0.001, respectively,); at 24 months, a significant decrease of BMD was detected only for LS (-2.5%, *P* < 0.001). However, 22% of patients became osteopenic and only 4% became osteoporotic.

**Conclusions:**

The addition of oral risedronate in post-menopausal breast cancer patients receiving anastrozole has a favorable effect on BMD. Patients with pre-treatment osteopenic to osteoporotic status should be treated with a combination of both therapies in order to avoid bone loss induced by aromatase inhibition. Patients with normal BMD before starting treatment with anastrozole have a very low risk to develop osteoporosis.

**Trial registration:**

ClinicalTrials.gov Identifier NCT00809484.

## Introduction

More than 70% of breast cancer patients develop endocrine-responsive disease with estrogen receptor (ER)-positive or progesterone receptor-positive tumors or both [1] and require endocrine treatment with either estrogen blockage or ablation. On the other hand, it is well known that estrogens have an indirect and positive effect on bone metabolism by stimulating the production of several cytokines acting either as inhibitors of osteoclastogenesis or as antireceptive agents leading active osteoclasts to apoptosis [2]. Therefore, the depletion of estrogens in patients with endocrine-responsive breast cancer leads to increased bone resorption and finally osteoporosis occurs, resulting in increased risk for bone fractures [3,4].

The introduction of aromatase inhibitors (AIs) during the last decade has opened new horizons in the successful treatment of ER-positive breast cancer. Clinical trials established the role of AIs in the adjuvant therapy of postmenopausal women with hormone-responsive breast cancer in upfront, switch, and sequential treatment settings [5] and this is reflected by international guidelines such as those of the American Society of Clinical Oncology [6], St. Gallen [7], the National Comprehensive Cancer Network [8], and others.

However, various clinical studies demonstrated that estrogen deprivation caused by AI administration has a serious negative effect on bone health [9]. Bone mineral density (BMD) rapidly decreases with a consequent high risk of skeletal fragility due to aromatase inhibitor-associated bone loss (AIBL). For the prevention of this adverse event, antiresoptive agents such as bisphosphonates (BPs) are used in combination with AIs.

BPs have a high affinity for hydroxyapatite, bind directly to mineralized bone, and enable the bone to be resistant to endogenous phosphatases [10]. On osteoclast stimulation of bone resorption, the BP is released and internalized by the osteoclasts, interfering with osteoclast formation, function, and survival [11]. Various compounds of BPs, available for either oral or intravenous administration, can have a beneficial effect on tumor-induced osteolysis, thereby minimizing the destructive consequences of estrogen deficiency-associated osteoporosis. However, oral BPs can be administered at home in weekly or monthly formulations, offering convenience for patients and, in this respect, could be the ideal treatment for the prevention of skeletal complications in early breast cancer patients with no evidence of metastatic spread to bones.

Arimidex Bone Mass Index and Oral Bisphosphonates (ARBI) is a phase III, multicenter, open-label clinical trial conducted by the Hellenic Society of Breast Surgeons. The primary aims of this study were to investigate the effect of risedronate on BMD changes from baseline in postmenopausal, early breast cancer patients receiving anastrozole with follow-up from baseline to 24 months and to evaluate the effect of anastrozole monotherapy on bone mass in a group of patients with normal BMD before starting treatment.

## Materials and methods

A total of 213 consecutive eligible postmenopausal patients who had histologically confirmed hormone receptor-positive breast cancer and who had completed primary surgery and chemotherapy (if indicated) and were scheduled to receive anastrozole were enrolled in the ARBI study. Patients were excluded if their menopause was induced by prior chemotherapy or any other drug therapy, and other exclusion criteria were evidence of metastatic bone disease by bone scans, previous hip (HP) fractures or prostheses, known bone metabolism disorder, non-treated hypocalcemia, and previous treatment with selective estrogen receptor modulators (SERMs), hormone-replacement therapy (HRT), or BPs and liver or renal dysfunction.

The primary endpoint of the study was to investigate the effect of risedronate in patients with mild osteopenia (randomized arms) receiving anastrozole therapy, measured in both lumbar spine (LS) and HP at 12 months, and the secondary endpoint was the investigation of this effect at 24 months. Other secondary endpoints were (a) to evaluate the effect of anastrozole on BMD in patients with normal BMD before starting treatment and (b) to investigate the effect of risedronate in patients receiving anastrozole therapy who have BMD in the region of severe osteopenia or osteoporosis.

Pre-treatment baseline BMD was evaluated at LS and HP by dual-energy x-ray absorptiometry (DEXA) and then was assessed at both sites at 12 and 24 months. All baseline and follow-up measurements of 170 patients (79.8%) were centrally performed in one referral center in Athens (type of absorptiometer: Explorer made by Hologic, Bedford, MA, USA). In 43 patients (20.2%), measurements were performed at a university hospital outside of Athens, using the same type and model of absorptiometer as well as the same software as those in Athens. All of the assessments were made by the same operator in both instances, and randomization was centrally performed for the entire population of the study in the referral center in Athens. All patients had to give informed consent prior to enrollment in the study. Full local ethics committee approval was successfully obtained in all sites recruiting patients for the study, and national ethics committee approval of the trial protocol was also obtained.

### Classification of patients and randomization

**Figure 1:**
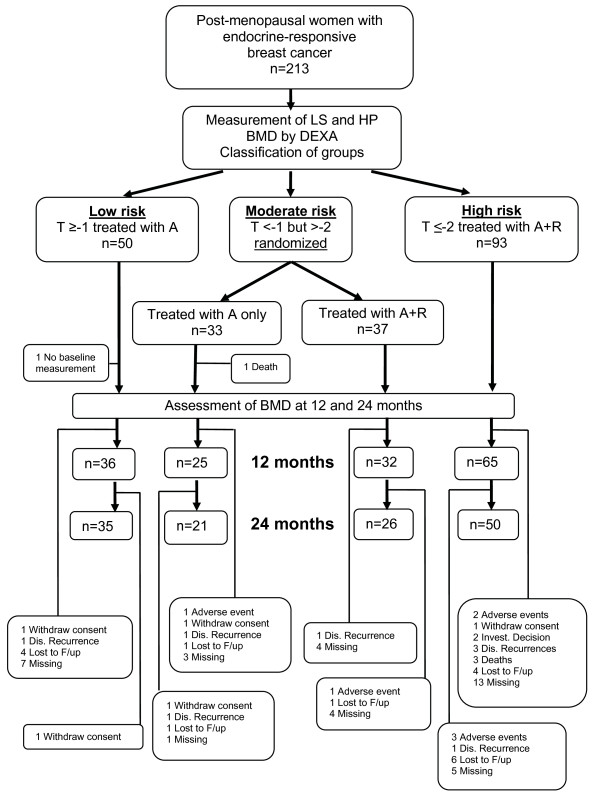
**Consort scheduled scheme of the ARBI (Arimidex Bone Mass Index and Oral Bisphosphonates) clinical trial**. The number of patients is included in data analysis. A, anastrozole; BMD, bone mineral density; DEXA, dual-energy x-ray absorptiometry; Dis., disease; F/up, follow-up; HP, hip; Invest., investigator; LS, lumbar spine; R, risedronate.

T-scores were determined according to the World Health Organization (WHO) definition as standard deviation (SD) units from the mean BMD of 25-year-old healthy women [12]. The BMD classifications, as defined by the WHO, were used to operationally define patient groups (normal = T-score of at least -1.0; osteopenia = -1 < T-score < -2.5; osteoporosis = T-score of not more than -2.5). In this trial, after baseline BMD measurement, patients were classified according to the relative risk of AIBL osteoporosis as follows: patients at low risk with a normal BMD T-score of at least -1 in both sites received anastrozole 1 mg/day (Arimidex™; AstraZeneca, London, UK) only; patients at mild to moderate risk with a BMD T-score of less than -1 in either site but a T-score of greater than -2.0 in both sites were randomly assigned to receive anastrozole 1 mg/day plus oral risedronate 35 mg/week (Actonel; sanofi-aventis, Paris, France) or anastrozole alone; patients at high risk with a T-score of not more than -2.0 in LS or HP received anastrozole 1 mg/day plus oral risedronate 35 mg/week. The classification of patients as described above is presented in Figure 1.

Risedronate tablets were taken once a week in accordance with instructions: it should be taken first thing in the morning with 100 mL of plain water at least 30 minutes before the first food or drink of the day, and to minimize the risk of esophageal irritation, the patient should not lie down or recline for at least 30 minutes. Patients were also advised not to eat or drink anything other than plain water or to take any other medicines, including vitamins, calcium, or antacids, for at least 30 minutes after taking Actonel. Additionally, all patients received daily supplements of calcium (1,000 mg/day) and vitamin D (400 IU/day) in accordance with the recommended guidelines of the American Society of Clinical Oncology [10]. BMD was then assessed at 12 and 24 months during scheduled follow-up visits. Throughout the study, the same densitometer was used to optimize the measurements.

### Statistical power and analysis

Since only two arms were randomized, in the moderate-risk patient subgroup, the design of the study was based on the expected difference between the two. The design parameters were based on information available from the 1-year results of the ATAC (Arimidex, Tamoxifen Alone or in Combination) study. In particular, in regard to the percentage change in BMD from baseline to 12 months for the anastrozole group in the LS and HP, the findings from the ATAC study were -2.26% change (interquartile range [IQR] -4.73% to -0.21%) and -1.51% change (IQR -3.16% to 0%), respectively.

Slight amplifications of these reductions (namely, 2.75% and 1.75% for the LS and HP, respectively) were used as the basis for the expected difference between the two randomized arms. The amplification was based on the hypothesis that a BMD increase for the risedronate arm should be expected and thus the difference would be larger than if we assumed that the risedronate arm would exhibit stable BMD values. For a baseline BMD value, we used the one that corresponds to a T-score of -1.5 (the median between the upper and lower limits defining the intermediate-risk group).

Using the assumption that the percentage change is a normally distributed variable, we estimated that the reductions in absolute terms would be 0.025 in the LS and 0.013 in the HP, with SDs of 0.031 and 0.018, respectively. The SD was calculated using the property of the normal distribution that the IQR = 1.34896 × SD.

A Bonferroni adjusted type I error rate of 2.5% for a double-two-sided *t *test with 80% power was used, and 36 patients were required for each arm under the more strict assumptions of the HP BMD expected differences (total 72) and 30 under the LS assumptions (total 60). With an anticipated 5% dropout rate, 38 patients should be recruited while 32 would suffice for the LS test. Study enrollment stopped when the required number of patients in the moderate-risk group was achieved.

A power of 79% for HP and of 86% for LS is achieved with the sample size of 57 patients available at 12 months, using the same assumptions as for the original design of the study and alpha = 0.05. When using Bonferroni adjustment, the corresponding power becomes 70% for HP and 78% for LS.

During analysis, the Wilcoxon and signed rank non-parametric tests were used for the univariate comparisons, and they were performed on the log-transformed values of percentage change from baseline to adjust for the presence of extreme values. In addition, mixed effects models were used to detect time trends and time by treatment interactions, taking into account both the observed LS and HP measurements at both 12 and 24 months. The mixed models technique uses all of the available patients regardless of the level of missing values at the course of time. Statistical analysis was conducted with SAS 9.1.3 statistical software (SAS Institute Inc., Cary, NC, USA).

## Results

A total of 213 patients entered the study between February 2005 and February 2007. Among them, 93 patients were classified as 'high-risk' patients, 70 as 'moderate-risk' patients, and 50 having normal BMD levels as 'low-risk' patients. Baseline characteristics are summarized in Table 1. On average, patients' baseline characteristics were similar among the treatment groups. ER status was positive for 89.3% of the patients in the high-risk group, 88.0% in the low-risk group, and 90.9% in the A-only randomized arm versus 97.3% in the A+R randomized arm. Similarly, progesterone receptor status was positive for 74.2% of the patients in the high-risk group, 80.00% in the low-risk group, and 72.7% in the A-only randomized arm versus 70.3% in the A+R randomized arm.

**Table 1:** Patients' baseline characteristics

	T ≤ -2, A+R (n = 93)		-2 < T < -1, A (n = 33)		-2 < T < -1, A+R (n = 37)		T ≥ -1, A (n = 50)	
	Mean	SD	Mean	SD	Mean	SD	Mean	SD
Age, years	65.7	7.8	64.5	9.2	62.6	8.5	62.0	7.7
Height, cm	158.3	6.7	154.2	28.0	156.6	27.0	161.7	5.4
Weight, kg	69.6	11.2	70.5	12.4	70.6	9.6	78.2	11.3
BMD LS value	0.75	0.10	0.93	0.10	0.99	0.11	1.10	0.10
BMD HP value	0.72	0.10	0.79	0.09	0.79	0.08	0.91	0.12
								
	**Number**	**Percentage**	**Number**	**Percentage**	**Number**	**Percentage**	**Number**	**Percentage**
								
ECOG status								
0	82	88.17	27	81.82	34	91.89	46	92.00
1	11	11.83	6	18.18	3	8.11	4	8.00
Fracture history								
No	86	92.47	32	96.97	35	94.59	44	88.00
Yes^a^	3	3.23	0	0.0	1	2.70	3	6.00
Not reported	4	4.30	1	3.03	1	2.70	3	6.00

^a^Traumatic fractures only; between 3 to 56 years before enrollment in the study; none in the hip (HP) or lumbar spine (LS). A, anastrozole; BMD, bone mineral density; ECOG, Eastern Cooperative Oncology Group; R, risedronate; SD, standard deviation; T, T-score.

**Figure 2:**
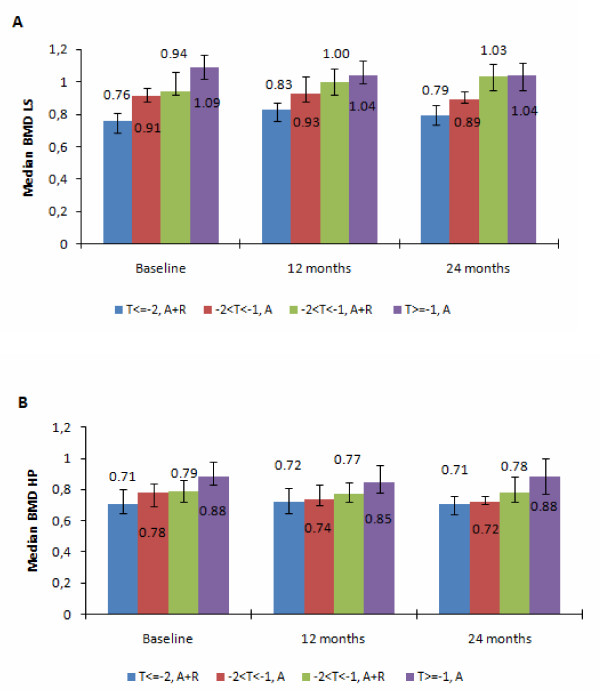
**Median bone mineral density (BMD) of lumbar spine (LS) (a) and hip (HP) (b)**. Error bars indicate interquartile range. A, anastrozole; R, risedronate.

In regard to BMD measurements, mean BMD values in LS were 0.75 for the high-risk group, 1.10 for the low-risk group, and 0.93 for the A-only randomized arm versus 0.99 for the A+R randomized arm. Mean BMD values in HP were 0.72 for the high-risk group, 0.91 for the low-risk group, and 0.79 for both the A-only and A+R randomized arms. Median BMD levels and IQR of measurements across all groups and across time are presented in Figure 2.

### Fracture history

Traumatic fracture history was present in seven patients: three in the high-risk (arm, wrist, and thoracic spine), three in the low-risk (ribs and clavicle, shin, and nose), and one in the A+R randomized (thoracic spine) arm (Table 1). However, in none of these cases did the fracture occur in the HP or LS; all fractures happened between 3 to 56 years before enrollment in the study.

### Comparison of randomized arms (A = 33 patients, A+R = 37 patients)

**Figure 3:**
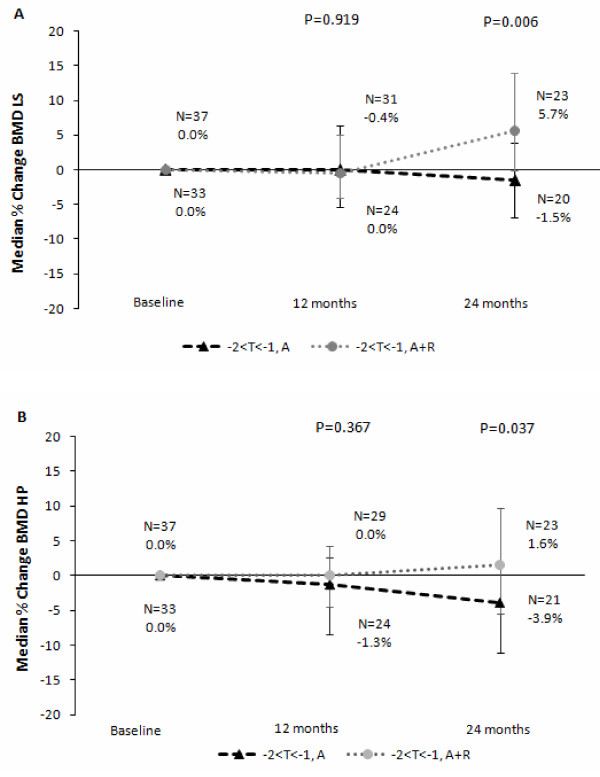
**Median change in bone mineral density (BMD) of lumbar spine (LS) (a) and hip (HP) (b)**. Error bars indicate interquartile range. A, anastrozole; R, risedronate (randomized arms only).

BMD value percentage change from baseline was significantly different for LS at 24 months (-1.5% for A versus 5.7% for A+R, Wilcoxon test *P *= 0.006; Figure 3) and was statistically significantly higher from baseline for the A+R arm (signed rank test *P *= 0.01; Table 2). For HP, a statistically significant decrease was observed at 24 months for the A arm (*P *value = 0.02) but not for the A+R arm (*P *value = 0.5) and was statistically significantly smaller for the A arm than the A+R arm (Wilcoxon test *P *value = 0.037; Figure 3). At 12 months, among A-only patients, 5 (15.2%) had a T-score of less than -2.0 without becoming osteoporotic whereas 2 (6.1%) moved to the normal BMD region; among A+R patients, only 2 (5.4%) had a T-score of less than -2.0 without becoming osteoporotic whereas 9 (24.3%) moved to the normal BMD region. The same trend in BMD changes was observed at the 24-month evaluation. It should be noted that the non-significant changes from baseline noticed at 12 months, should be assessed in light of the fact that the power levels achieved with the 57 patients at 12 months are 70% for the comparisons involving HP and 78% for the comparisons involving LS.

**Table 2:** Descriptive statistics of bone mineral density lumbar spine and hip percentage change from baseline at 12 and 24 months

		BMD lumbar spine percentage change from baseline				BMD hip percentage change from baseline			
		T ≤ -2, A+R	-2 < T < -1, A	-2 < T < -1, A+R	T ≥ -1, A	T ≤ -2, A+R	-2 < T < -1, A	-2 < T < -1, A+R	T ≥ -1, A
12 months	Median	6.3%	0.0%	-0.4%	-5.3%	-1.9%	-1.3%	0.0%	-2.4%
	IQR	12.0%	12.0%	9.2%	8.2%	11.0%	11.0%	8.6%	6.7%
	*P *value	< 0.001	0.70	0.68	< 0.001	0.30	0.22	0.76	< 0.001

24 months	Median	6.6%	-1.5%	5.7%	-2.5%	-1.9%	-3.9%	1.6%	-5.7%
	IQR	17.0%	11.0%	14.0%	10.0%	19.0%	12.0%	15.0%	13.0%
	*P *value	< 0.001	0.25	0.01	< 0.001	0.16	0.02	0.50	0.09

A, anastrozole; BMD, bone mineral density; IQR, interquartile range; R, risedronate; T, T-score.

In the randomized arms, an analysis of mixed models examined the percentage change from baseline in HP and LS BMD values. The analyses were adjusted for the baseline value of the outcome variable and time. In the case of the percentage change in HP BMD value, only the baseline value of BMD was statistically significant (Table 3). In the case of the percentage change in LS BMD value, the interaction of treatment arm with time is statistically significant (Table 3, *P *= 0.004), and there is a statistically significant effect of BMD value at baseline (Table 3, *P *= 0. 005). In regard to the significance of interaction, it means that the treatment effect on BMD LS is not constant across time. In particular, at 12 months, the average change of the A+R arm is only 1.8% larger than the change of the A arm (Table 3) and this is not statistically significant (*P *= 0.506), whereas at 24 months, the average change of the A+R arm is (1.8% + 9.0% = 10.8%) larger than the change of the A arm, and this increment (9.0%), which increases the difference between the two arms, is statistically significant (*P *= 0.004). In regard to the negative effect of the baseline values, it implies that higher baseline BMD values correspond to smaller changes at both 12 and 24 months whereas patients' smaller BMD values at baseline exhibited a higher trend of BMD increase.

**Table 3:** Mixed models for percentage change of bone mineral density from baseline

Effect	Estimate	Standard error	*P *value
Hip			

Treatment arm^a^	0.042	0.022	0.069

Time	0.004	0.022	0.838

BMD value at baseline	-0.446	0.131	0.001

Lumbar spine			

Treatment arm^a, b^	0.018	0.027	0.506

Time^c^	-0.035	0.022	0.122

Time × treatment arm^d^	0.090	0.030	0.004

BMD value at baseline	-0.328	0.113	0.005

^a^Reference category is treatment arm A (A+R versus A). ^b^Treatment arm effect at 12 months. ^c^Time effect for arm A. ^d^Treatment arm effect at 24 months. A, anastrozole; BMD, bone mineral density; R, risedronate.

### Group with T-score of not more than -2.0 in either lumbar spine or hip (A+R, n = 93)

A significant increase for LS at both 12 and 24 months was detected (median increase of BMD by 6.3% and 6.6%, respectively, *P *< 0.001 for both time points; Table 2) with a corresponding non-significant change in HP (-1.9% median change of BMD value at 12 months, *P *= 0.30 and median decrease of BMD value by -1.9%, *P *= 0.16 at 24 months; Table 2). BMD in 13 patients (14%) improved to the osteopenic region.

### Group with T-score of at least -1 in both sites (A, n = 50)

A significant decrease was observed for LS at both 12 and 24 months (median decrease of BMD value by -5.3% and -2.5% for LS, *P *< 0.001 for both time points; Table 2). For HP, a significant reduction was observed at 12 months but was only marginally significant at 24 months, probably due to the large between-patient variation (IQR = 13% at 24 months versus 6.7% at 12 months) (median decrease of BMD by -2.4%, *P *< 0.001 and -5.7%, *P *= 0.09, respectively); however, only 11 patients (22%) became osteopenic and 2 (4%) became osteoporotic. In regard to the interpretation of the non-significant differences in the non-randomized arms, it should be noted that the study was not designed to detect differences in this respect and may be underpowered. However, the finding of significant differences is an indication of sufficient power to detect differences of the size observed.

### Adverse events

Due to adverse events, seven patients stopped treatment (Figure 1): one in the A arm and one in the A+R randomized arm (severe allergic skin reaction and severe myalgia, respectively, most likely due to anastrozole) and five patients in the A+R high-risk group (upper gastrointestinal tract symptoms attributed to oral BPs). Additionally, eight more patients among those under risedronate treatment (6%) experienced mild gastrointestinal tract symptoms such as nausea and indigestion and 14 patients (6.6%) suffered known anastrozole mild adverse events, 50% of which were joint pains (two patients in each of the randomized arms, two patients in the normal BMD region, and one osteoporotic patient). None of the remaining events appeared more than once, and none of the 14 adverse events was considered serious. No fragility fractures were reported in any group of patients during the study, and no case of osteoporosis of the jaw was observed in any patient under risedronate treatment. Compliance to treatment was confirmed during the interview in each patient's follow-up visit; occasionally, missed tablets of either anastrozole or risedronate were reported by less than 10% of the patients under study treatment.

## Discussion

The evolution of adjuvant endocrine therapy contributes to the survival of postmenopausal hormone-related early breast cancer patients. The use of AIs changed the initial treatment with the previous gold standard of tamoxifen, as the comparison between them and tamoxfen emerged the efficacy of AIs in ER-dependent breast tumors' pharmacological strategies. However, as accelerated bone loss is associated with estrogen deficiency, AIBL is a very frequent complication. Osteoporosis can be developed and is amplified by age-related lack of estrogens, which increases the risk of vertebral and HP fractures.

The ATAC clinical trial was the first to show that the AI anastrozole was more effective than tamoxifen as first-line adjuvant hormonal therapy for early-stage breast cancer [13-15]. In the prospective bone subprotocol of the ATAC trial (n = 308), 2 years of anastrozole significantly reduced LS BMD by 3.97% and total HP BMD by 3.92% compared with tamoxifen [16]. After 5 years, 17% of baseline normal BMD patients receiving anastrozole became osteopenic. Similar changes were found in our study. We have also observed bone loss in patients treated with anastrozole without BP therapy (non-randomized arm A and randomized arm A) in both sites, LS and HP, over time. Among patients with normal BMD before starting treatment, 11/50 (22%) became osteopenic but only 2/50 (4%) became osteoporotic, while in the osteopenic group of patients, the T-score was further decreased in 5/33 cases (15.2%) but no patient became osteoporotic.

Letrozole is also associated with an increase in fracture risk, and in the Breast International Group (BIG) 1-98 clinical trial [17], a 40% excess of fractures was observed in the letrozole-treated patients (8.6% versus 5.8% for tamoxifen). Hadji and colleagues [18], in the German bone substudy of the Tamoxifen Exemestane Adjuvant Multicenter (TEAM) clinical trial, investigated the effect of treatment with exemestane on bone health and observed an increase in bone loss at 6 months compared with tamoxifen; bone loss was then stabilized after 6 to 12 months of treatment. Furthermore, the ALIQUOT (Anastrozole versus Letrozole Investigation of Quality Of Life and Tolerability) study showed that prior treatment with tamoxifen profoundly increases the effects of AIs on bone turnover and that these effects increase over time. Major increases in bone turnover are seen when tamoxifen is stopped and then followed by anastrozole or letrozole administration [19].

Bone health is clearly an important concern for breast cancer patients and, before the start of treatment, needs to be evaluated by oncologists by using baseline DEXA scanning and known clinical risk factors such as family history, cigarette smoking, and excessive alcohol consumption. Specific guidelines on how to evaluate and manage cancer therapy-induced bone loss were recently published by Hadji and colleagues [20]. AI use is a major additional cancer treatment-related risk factor in postmenopausal breast cancer patients. However, our findings along with data from the ATAC study and the Intergroup Exemestane Study (IES) [16,21] indicate that women with normal BMD before starting endocrine therapy have a very low risk of developing osteoporosis and that only the use of general preventive measures for maintaining bone health in postmenopausal women seems to be appropriate practice. Nevertheless, a lot of patients will still have established osteopenia or osteoporosis and need some other intervention to minimize their risk of ongoing loss of bone density due to long-term AI treatment.

Prevention of continuously decreasing BMD during endocrine treatment with AIs can be achieved with the appropriate administration of BPs. Several clinical trials demonstrate that the combination of AIs with BPs has a potent effect on BMD. The Austrian Breast and Colorectal Cancer Study Group trial-12 (ABCSG-12) bone substudy assessed zoledronic acid for preventing bone loss during adjuvant endocrine therapy [22]. The investigators concluded that hormonal treatment for 3 years without concomitant zoledronic acid caused significant bone loss at the LS and trochanter (-11.3% and -7.3%, respectively) and that the administration of BP improved BMD (LS +4.0% and trochanter +3.9%) compared with baseline at 5 years. In three Zometa-Femara Adjuvant Synergy Trials (Z-FAST, ZO-FAST, and E-ZO-FAST), patients received letrozole therapy combined with either immediate or delayed (that is, after a fracture or after BMD T-score decreased to -2.0) zoledronic acid treatment [23-25]. Patients who have been administrated immediately with zoledronic acid treatment had significant increases in BMD and had fewer fractures overall than patients who have delayed treatment (*P *< 0.0001 for all).

Considering oral administration of BPs, Lester and colleagues [26], in the ARIBON (Arimidex Bondronate) study, reported that, after 2 years, osteopenic patients treated with monthly doses of oral ibandronate gained +2.98% (range -8.9 to +19.9) and +0.60% (range -9.0 to +6.9) at the LS and HP, respectively. Patients treated with placebo, however, lost -3.22% (range -16.0 to +4.3) at the LS and -3.90% (range -12.3 to +7.2) at the HP. Additionally, a recently published report of the Study of Anastrozole with the Bisphosphonate Risedronate (SABRE) reported that oral risedronate 35 mg weekly results in favorable effects in BMD [27]. The SABRE study demonstrated that, in postmenopausal women who are at risk of fragility fracture and who are receiving adjuvant anastrozole, the addition of risedronate led to a 1% to 3% increase in LS BMD and a 1% to 2% increase in total HP BMD during a period of 24 months. Bone turnover markers were also measured and found to be suppressed by 3 months in patients receiving risedronate [27]. Our findings are in agreement with data from these studies. The addition of 35 mg oral risedronate weekly to anastrozole treatment in osteopenic patients (randomized arm A+R) resulted in a significant increase in BMD. Moreover, in the non-randomly assigned group of patients (T-score of not more than -2.0) who were all treated with risedronate, a significant increase for BMD at LS was detected, with a number of patients moving from osteoporotic to the osteopenic region. However, the statistically significant increase for BMD at HP found in the SABRE trial at 24 months was not detected in this non-randomly assigned arm in our trial, showing a mild numerical decrease at the same time point.

All of the above data support the common perception that bone protection with a BP is required for patients with osteoporosis. However, there is still a controversy on the management of patients with osteopenia. Among our osteopenic group of patients (randomized arm A) receiving additionally only daily supplements of calcium and vitamin D, the T-score became less than -2 in only 5/33 cases (15.2%), but no patient became osteoporotic (T-score of less than -2.5) during the 2 years of our study. On the other hand, the addition of 35 mg oral risedronate weekly to anastrozole treatment in osteopenic patients (randomized arm A+R) resulted in a significant increase in BMD, with 9/37 patients (24.3%) moving to the normal BMD region. In the medical community, a healthy lifestyle and adequate intake of calcium and vitamin D, along with 12 to 24 months of evaluation of BMD by DEXA, are acceptable ways of managing this group of patients with mild to moderate osteopenia. However, data from a number of studies and our findings presented here indicate that BPs might also be used in order to prevent further bone loss in this patient population.

Overall tolerability of oral administration of BPs in our study was good, with only five patients (4%) stopping treatment with risedronate due to upper gastrointestinal tract symptoms and eight more patients (6%) experiencing mild symptoms such as nausea and indigestion. No case of osteonecrosis of the jaw (ONJ) was observed in any patient under risedronate treatment. Notably, long-term BP therapy is associated with ONJ, although its estimated frequency in patients taking oral BPs for osteoporosis is less than 1 case per 100,000 person-years of exposure [28].

Finally, there is also a fascinating trend toward the potential adjuvant benefit of BPs in the improvement of clinical outcome of patients with early-stage breast cancer [29-31] and the reduction of breast cancer risk as well [32,33]. However, it is too early to consider BPs either in the prevention or in the adjuvant treatment setting until more data become available from large prospective trials [34].

## Conclusions

Our findings suggest that patients with normal BMD before starting treatment with anastrozole have a very low risk to develop osteoporosis during the first 2 years of treatment; general preventive measures such as healthy lifestyle and daily supplements of calcium and vitamin D seem to be adequate treatment for retaining bone health in this group of patients, which has a 20% chance to develop osteopenia only. On the other hand, the addition of oral risedronate in postmenopausal breast cancer patients in the high-risk region (T-score of not more than -2.0) receiving anastrozole has a significant increase in BMD levels. Bone protection with a BP is certainly indicated for patients with established osteoporosis. For patients at mild to moderate risk (-2.0 < T-score < -1), the combination of both therapies (anastrozole plus oral risedronate) might also be used in order to prevent bone loss.

## Abbreviations

A: anastrozole; AI: aromatase inhibitor; AIBL: aromatase inhibitor-associated bone loss; ARBI: Arimidex Bone Mass Index and Oral Bisphosphonates; ATAC: Arimidex, Tamoxifen Alone or in Combination; BMD: bone mineral density; BP: bisphosphonate; DEXA: dual-energy x-ray absorptiometry; ER: estrogen receptor; HP: hip; IQR: interquartile range; LS: lumbar spine; ONJ: osteonecrosis of the jaw; R: risedronate; SABRE: Study of Anastrozole with the Bisphosphonate Risedronate; SD: standard deviation; WHO: World Health Organization.

## Competing interests

CM has received educational grants and lecture honoraria from AstraZeneca, Novartis (Basel, Switzerland), and Pfizer Inc (New York, NY, USA). ET, AP, BV, GX, JP, VZ, JM, KK, DK, ZA, and HG have received unrestricted educational grants from AstraZeneca, Novartis, and Pfizer Inc. The other authors declare that they have no competing interests.

## Authors' contributions

CM conceived, designed, and coordinated the study, provided study material, and drafted the manuscript. UD coordinated the study, performed the statistical analysis, and helped to draft the manuscript. ET, AP, BV, GX, JP, VZ, JM, KK, AS, NS, DK, and ZA participated in the design of the study and provided study material. SL participated in the design of the study and performed the DEXA measurements. HG participated in the design of the study, provided study material, and helped to draft the manuscript. All authors read and approved the final manuscript.
